# Editorial: Anthropogenic wildlife movements and infectious diseases: Health and conservation perspectives

**DOI:** 10.3389/fvets.2023.1132176

**Published:** 2023-03-07

**Authors:** David González-Barrio, Mathieu Pruvot, Richard Anthony Kock, Xavier Fernández Aguilar

**Affiliations:** ^1^Parasitology Reference and Research Laboratory, Spanish National Centre for Microbiology, Health Institute Carlos III, Madrid, Spain; ^2^Wildlife Conservation Society, Health Program, The Bronx, NY, United States; ^3^Department of Pathobiology and Population Sciences, Royal Veterinary College, London, United Kingdom; ^4^Department of Ecosystem and Public Health, Faculty of Veterinary Medicine, University of Calgary, Calgary, AB, Canada

**Keywords:** anthropogenic, wildlife movement, infectious diseases, conservation, translocation, pathogens

Animal movements due to human activities are the cause of local, regional, national and international spread of animal diseases, including pathogens of public health concern (1, 2). Anthropogenic movements of wildlife can be due to several reasons: (i) restocking of game animals for hunting purposes, (ii) translocations or reintroductions in the framework of biodiversity conservation programs and population managements, (iii) national and international trade (both legal and illegal), but also a range of (iv) human activities including land use and alterations of habitats that can indirectly influence wildlife movements and interactions (e.g., habitat fragmentation and encroachment). Disease threats associated with these wildlife movements include the introduction of exotic pathogens, changes in epidemiological patterns and risks of cross-species transmission or recurrence in areas previously free. Understanding these disease risks is necessary to increase awareness and facilitate preventive and control measures to reduce disease spread and further conservation threats.

The overall aim of this Research Topic is to collect relevant past or current information illustrating how anthropogenic wildlife movements could result in the spread of disease and provide examples on how this risk has been managed.

In this Research Topic, Chaber et al., identified in a preliminary study that thousands of bat specimens belonging to 32 different bat species from 24 countries were traded online during 2 weeks in May 2020. Specimens of these bats included species listed as threatened and included in the CITES Appendix II. This trade and movement of animals could have consequences on disease spread and be a threat to conservation and biodiversity due to the important role of bats in ecosystems. Current international legislation regulates the legal trade of wildlife and provides a barrier to entry of endangered species and CITES-listed animals. These regulations include bat species, but their enforcement is a challenge due to the sheer volume of items being traded on the internet and shipped daily. The study also highlights that this trade may be associated with the spread of zoonotic pathogens, as some species of bat are reservoirs of important zoonotic viruses like ebolaviruses, coronaviruses and lyssaviruses, which can remain temporarily stable under variable conditions. It is important that we improve surveillance and our understanding of the risks associated with wildlife trade, specifically the electronic bat trade, in terms of viral transmission, risks of spillover and how to adjust current social networking and cultural practices to mitigate zoonotic risks.

Ryser-Degiorgis et al. describe the management of suspected cases of an infectious disease in the framework of an international translocation program on Eurasian lynx (*Lynx lynx*). During 2016–2017, some captured lynx from the same geographical area were found seropositive for feline immunodeficiency virus (FIV). This detection raised questions about the origin of the infection as well as the pathogenicity of this virus to the lynx. The seropositive lynx were monitored in quarantine enclosures and were euthanized because they developed clinical signs. Pathological findings and occurrence of co-infections were similar to those described in domestic cats with FIV. Although the virus could not be isolated or characterized, the serological data and the spatio-temporal proximity of the cases suggested that the infection could be due to the emergence of a lentivirus in the Swiss lynx population with antigenic and pathogenic similarities to FIV. Thus, a decision scheme was developed to minimize the potential health risks posed by FIV infection in both the recipient and source populations of lynx, considering conservation, welfare and disease risk implications. The decision scheme included three different scenarios: (i) release at the capture site, (ii) translocation or (iii) euthanasia depending on bite wounds, evidence of viraemia and exposure or signs of disease. Development and implementation of the decision scheme in subsequent captures was made difficult by the uncertainty of the pathogenic potential of the virus and possible false-negative serological results during the first weeks of infection. This article provides a useful case study on the complexity of decision making in a wildlife translocation program, in which decisions must weigh disease risks and conservation interests.

Sherman et al., have also studied the importance of these translocations in endangered wildlife. In this case, translocated orangutans that are exposed to human diseases, such as COVID-19, pose a health risk to both wild and previously released individuals. Wildlife disease risk experts advised that movements of great apes should be halted for the duration of the COVID-19 pandemic to minimize the risk of disease transmission to wild populations. The authors collected data on orangutan releases and associated disease risk management in Indonesia during the COVID-19 pandemic, and developed a problem description of orangutan disease and conservation risks. Disease risks were identified in orangutans translocated both from wild to wild and in orangutans rehabilitated in captivity that have had long periods of contact and potential exposure to human diseases. These risks were due to direct contact or proximity to humans without protective equipment. COVID-19 and other human-borne diseases can be transmitted to orangutans, which could have catastrophic implications for wild orangutans, other vulnerable wildlife and humans in the event of disease transmission. The authors recommended conducting a disease risk analysis for orangutan translocation and improving pathogen surveillance as well as implementing mitigation measures to reduce the potential for outbreaks. They also suggested redirecting conservation efforts toward alternatives to wild-to-wild translocation, such as mitigating human-orangutan interactions, enforcing laws and protecting orangutan habitats to improve *in-situ* conservation.

Finally, Ebhodaghe et al., presents research on vector-borne pathogen transmission in anthropised landscapes. Shimba Hills is a wildlife area in Kenya and a major focus of tsetse-borne trypanosomes in East Africa. In Shimba Hills, tsetse-transmitted trypanosomes are detrimental to domestic animals health and the livelihoods of smallholder farmers. However, there are no epidemiological data to guide infection control in both wildlife and domestic livestock hotspots. This study assessed the risk of tsetse-transmitted trypanosomes in the Shimba Hills with the aim of understanding drivers of disease outbreaks for its prevention and control. The authors concluded that cattle at Shimba Hills are at high risk of trypanosome infection from female tsetse and *G. pallidipes* flies in grazing fields near the wildlife reserve. This study shows that the tsetse fly exists at high infestation levels near the wildlife reserve, due to favorable living conditions and the likelihood of vectors feeding on wildlife in these locations. Livestock is potentially exposed to infections from wild reservoirs of trypanosomes.

Overall, the articles in this Research Topic illustrates some aspects of how anthropogenic wildlife movements may lead to the spread of pathogens, from applied field studies to investigations on the illegal trade of species, highlighting the complex interaction between pathogens, wildlife, livestock and humans. This topic provides useful examples of human-altered processes of disease spillover between hosts and emergence.

It should be emphasized that limited data is often available on the health of specific wildlife populations, limiting our ability to fully measure and understand the effects of human-induced wildlife movements on pathogen spread and dynamics, and their downstream effect on wildlife populations and conservation. However, disease risk can be identified, assessed, and minimized using structured frameworks. Guidelines for wildlife disease risk analysis are provided by the International Union for the Conservation of Nature (IUCN) and the World Organization for Animal Health (OIE/WOAH) (3), or specific research groups [e.g., (4)], to manage and minimize these risks in the context of purposive anthropogenic wildlife movements. The importance of implementing these frameworks is stressed by the catastrophic consequences of the introduction of infectious diseases following wildlife anthropogenic movements (e.g., Myxomatosis in lagomorphs from the Mediterranean area, squirrelpox in squirrels from UK or afanomycosis in the European freshwater crayfish). We should learn from these previous experiences and implement measures to minimize these disease risks from anthropogenic wildlife movements. However, intentional wildlife movement is only a fraction of anthropogenic wildlife movements, as land-use change, habitat degradation, climate change, alteration of migratory processes continue to alter wildlife distribution and movement patterns. Better connecting wildlife health monitoring with climate adaptation, wildlife management (5), and environmental risk assessment (6), will continue to be essential in better assessing the effects of anthropogenic wildlife movement on infectious diseases and their impact on wildlife populations.

## Author contributions

All authors listed have made a substantial, direct, and intellectual contribution to the work and approved it for publication.
